# Early allogeneic immune modulation after establishment of donor hematopoietic cell-induced mixed chimerism in a nonhuman primate kidney transplant model

**DOI:** 10.3389/fimmu.2024.1343616

**Published:** 2024-01-22

**Authors:** Christopher J. Little, Steven C. Kim, John H. Fechner, Jen Post, Jennifer Coonen, Peter Chlebeck, Max Winslow, Dennis Kobuzi, Samuel Strober, Dixon B. Kaufman

**Affiliations:** ^1^ Department of Surgery, University of Wisconsin School of Medicine & Public Health, Madison, WI, United States; ^2^ Department of Surgery, University of Washington School of Medicine, Seattle, WA, United States; ^3^ Department of Surgery, Emory University School of Medicine, Atlanta, GA, United States; ^4^ Wisconsin National Primate Research Center, University of Wisconsin, Madison, WI, United States; ^5^ Department of Medicine, Stanford University School of Medicine, Palo Alto, CA, United States

**Keywords:** mixed chimerism, kidney transplant, PD-1, Tregs, nonhuman primate

## Abstract

**Background:**

Mixed lymphohematopoietic chimerism is a proven strategy for achieving operational transplant tolerance, though the underlying immunologic mechanisms are incompletely understood.

**Methods:**

A post-transplant, non-myeloablative, tomotherapy-based total lymphoid (TLI) irradiation protocol combined with anti-thymocyte globulin and T cell co-stimulatory blockade (belatacept) induction was applied to a 3-5 MHC antigen mismatched rhesus macaque kidney and hematopoietic cell transplant model. Mechanistic investigations of early (60 days post-transplant) allogeneic immune modulation induced by mixed chimerism were conducted.

**Results:**

Chimeric animals demonstrated expansion of circulating and graft-infiltrating CD4+CD25+Foxp3+ regulatory T cells (Tregs), as well as increased differentiation of allo-protective CD8+ T cell phenotypes compared to naïve and non-chimeric animals. *In vitro* mixed lymphocyte reaction (MLR) responses and donor-specific antibody production were suppressed in animals with mixed chimerism. PD-1 upregulation was observed among CD8+ T effector memory (CD28-CD95+) subsets in chimeric hosts only. PD-1 blockade in donor-specific functional assays augmented MLR and cytotoxic responses and was associated with increased intracellular granzyme B and extracellular IFN-γ production.

**Conclusions:**

These studies demonstrated that donor immune cell engraftment was associated with early immunomodulation via mechanisms of homeostatic expansion of Tregs and early PD-1 upregulation among CD8+ T effector memory cells. These responses may contribute to TLI-based mixed chimerism-induced allogenic tolerance.

## Introduction

Allogeneic immunoreactivity is a major barrier to the long-term survival of donor grafts following solid organ transplantation. The best therapeutic regimens of systemic immunosuppression make transplantation possible by effectively diminishing host allo-immune responses; however, there is cost, morbidity, and mortality associated with these medications. Induction of donor-specific tolerance, a state of allogenic immune unresponsiveness, obviates the need for lifelong immunosuppression (IS) and its adverse effects, representing the ideal post-transplant state.

Establishing mixed lymphohematopoietic chimerism that is comprised of recipient- and donor-derived cellular immune elements has emerged as a proven strategy for the induction of allograft tolerance (1–16). A novel tolerance induction protocol developed at Stanford University involving a post-transplant, non-myeloablative, total lymphoid irradiation (TLI) and anti-thymocyte globulin (ATG) conditioning regimen has been reported to achieve engraftment of donor hematopoietic cells (HCs). This protocol has been successful in human clinical trials of combined kidney and HC transplantation between HLA-identical pairs (2–4). We have previously shown that this protocol, modified to utilize helical Tomotherapy-based TLI (TomoTLI), is capable of achieving transient mixed chimerism-based operational tolerance between more disparate major histocompatibility complex (MHC) donor-recipient pairs in a 1-haplotype matched rhesus macaque model, permitting complete withdrawal of IS with >4 years of graft survival (17).

Unlike a strategy of total chimerism, transient mixed chimerism, mitigates the risk of graft-versus-host disease (GVHD), highlighting an important safety feature of this tolerance induction strategy. The Stanford human protocol requires stable mixed chimerism to prevent rejection after IS withdrawal; however, several tolerance induction studies have demonstrated the sufficiency of transient mixed chimerism for IS-free survival of renal allografts (2, 11–13). This distinction is driven by the development of complex multifactorial peripheral mechanisms of donor-specific immunomodulation, including induction of T cell anergy and exhaustion, expansion of regulatory T cells (Tregs), and peripheral differentiation of allo-protective T cell phenotypes (18, 19). However, despite an important body of research exploring these mechanisms, the underpinnings of allogenic tolerance induction during the establishment of mixed chimerism remain incompletely understood. Further, the necessary level and duration of chimerism to induce these immunologic changes has yet to be elucidated.

We have previously demonstrated in a 1-haplo-matched rhesus macaque model that a post-transplant, non-myeloablative, TomoTLI-based conditioning regimen followed by donor HC infusions can induce transient mixed chimerism and subsequent allogenic tolerance (17). Given these promising findings, we applied the foundations of this previously reported conditioning protocol to a 3-5 antigen mismatched rhesus kidney transplant tolerance induction model, with protocol modifications to mitigate the development of early DSA (20–23). Specifically, belatacept, a costimulation blockade agent targeted against the CD28-B7 pathway was added to the treatment regimen, as well as an extended steroid taper to reduce the incidence of engraftment syndrome, a major barrier to kidney survival among previous 1-haplotype recipients (24–27). This report provides important mechanistic insights into the early immunologic effects of low-level, transient mixed chimerism induction using non-myeloablative TomoTLI-based conditioning.

## Materials and methods

### Animal characterization, typing, and determination of donor-recipient pairs

Rhesus macaques were obtained from the National Institute of Allergy and Infectious disease colony maintained by Alpha Genesis Inc. (Yemassee, SC) as previously described (17, 28). All animals were treated in accordance with the 8^th^ edition of the Guide for the Care and Use of Laboratory Animals published by the National Research Council. All procedures and protocols were approved by the University of Wisconsin-Madison Institutional Animal Care and Use Committee.

At the time of transplant, animals were 5.3-12.2 kg and 4.5-5.1-years-old. Both males and females were used as donor and recipients. MHC Class I and Class II typing were performed by the Wisconsin National Primate Research Center (WNPRC) and its Genetics Service Unit as previous described (17, 29, 30). Donor and recipient pairs were selected based on MHC haplotyping results to arrange for maximal MHC antigen disparity.

### Post-transplant mixed chimerism-based tolerance induction protocol

A post-transplant, TomoTLI and ATG conditioning regimen was applied to a rhesus macaque kidney transplant tolerance model as previously described (17, 28). Prior to conditioning, recipient macaques underwent solitary allogenic kidney transplantation (day 0) and bilateral native nephrectomies (submitted to pathology). Induction therapy was initiated on day 0 with five consecutive daily doses of 4mg/kg rhesus-specific ATG (NIH Nonhuman Primate Reagent Resource, Boston, MA) as previously described (17, 28). Belatacept 10mg/kg (Bristol-Myers Squibb, Princeton, NJ) was infused on days 11, 14, and 18.

TomoTLI was delivered as previously reported (17, 28). Briefly, starting on day 1, ten fractions of TLI (1.2 gray/fraction) were delivered by image-guided, intensity modulated helical tomotherapy (TomoTherapy Hi-Art II, Accuracy Inc, Sunnyvale, CA) to total a cumulative dose of 12 gray (Gy). The total lymphoid target included the inguinal, iliac, sublumbar, para-aortic, axillary and mandibular lymph nodes, as well as the spleen and anterior mediastinal/thymic tissues. Offline adaptive planning was used to account for changes in body weight or composition during radiation delivery.

Donor-derived bone marrow (BM) infusion was performed on day 15. Donor animals received three daily doses of granulocyte colony-stimulating factor (G-CSF) (100mcg/kg) prior to collection. Deceased donor BM procurement occurred after confirmation of donor death by the veterinary team. Bilateral femurs and humeri were explanted, segmented, and flushed with heparinized saline to collect the marrow compartment. The BM was filtered through a 100-micron strainer to isolate the cellular component. Flow cytometry was performed on a small aliquot to determine the CD34+ and lineage-committed composition of the infusion product.

General anesthesia was utilized for all operative procedures and delivery of TomoTLI. Induction and maintenance of anesthesia, as well as endotracheal intubation and ventilator management, was performed by trained members of the WNPRC veterinarian team. Animals were sedated with ketamine (5-15mg/kg IM) and midazolam (0.2mg/kg IM) prior to intubation. Anesthesia was then maintained with isoflurane gas (0.5-3%) or serial doses of ketamine (5-15mg/kg IM) and dexmedetomidine (0.015mg/kg IM) and was reserved with atipamezole (0.015mg/kg IM). Port placement was performed under moderate sedation with meloxicam (0.2mg/kg SQ) and buprenorphine (0.01-0.03 mg/kg IM). Euthanasia was performed humanely by first sedating the animal with ketamine (>15mg/kg IM) followed by administration of sodium pentobarbital (>50mg/kg IV).

### Maintenance immunosuppression

Maintenance IS consisted of a prednisone taper (2mg/kg/day tapered over 84 days) and mycophenolate mofetil (MMF) 15mg/kg per mouth daily as well as tacrolimus intramuscularly (IM) BID and sirolimus IM daily, with levels monitored 1-2 times per week to maintain trough levels of 8-10ng/mL and 2-4ng/mL, respectively. At day 90, MMF was scheduled to be tapered by 25% every two weeks for discontinuation on day 132. Sirolimus was to be eliminated on day 132. Tacrolimus taper was to begin two weeks after MMF/sirolimus-cessation by reducing the trough target by 25% per month until discontinuation on day 224. All recipients received a course of prophylactic antibiotics as previously reported (17, 28).

### Experimental reagents and design

All *in vitro* reagents were obtained from the NIH Nonhuman Primate Reagent Resource or were available from commercial biotechnology companies. Information regarding antibody panels, clones and reagent manufacturers is available in **Supplementary Table 1**. Antibody stains were used at the manufacturer’s recommended concentration. All flow cytometric data was acquired on a BD LSR II flow cytometer instrument and analyzed using FlowJo Software (Ashland, OR).

### Chimerism testing, T cell immunophenotyping, and histology analysis

All transplant pairs were mismatched for at least one MHC Class I allele, such that recipients were Mamu-A*01 negative and donors were Mamu-A*01 positive to allow for flow cytometric analysis of lineage-specific peripheral chimerism utilizing an anti-Mamu-A*01 antibody. Whole blood was collected weekly following donor cell infusion and stained for Mamu-A*01, as well as common leukocyte surface markers.

Peripheral blood mononuclear cells (PBMC) were isolated from recipients prior to transplant, as well as at monthly time points following donor cell infusion. Spleen, lymph nodes, and kidney allograft were recovered and processed for lymphocytes at the time of necropsy. Surface and intracellular staining were performed with antibodies against T cell phenotypic markers.

Allograft tissue was fixed in formalin and sectioned by pathology for hematoxylin and eosin, periodic acid-Schiff, trichrome, and C4d staining. Analysis was performed and reported by experienced renal pathologists with expertise in transplant allograft histology.

### Functional *in vitro* assays

Allogenic mixed lymphocyte reactions (MLRs) were performed using fresh PBMC collected from recipients prior to transplant and between post-infusion day (PID) 30-60, which were stimulated with donor leukocytes (thawed from cryopreservation). Negative, autologous controls were set up against leukocytes derived from the recipient. Responder cells were labeled with Cell Trace Violet (CTV), while stimulators were separately labeled with Cell Trace Far Red (CTFR). Stimulators were irradiated (20 Gy) prior to plating. A 4-day co-culture was set up using 2.0x10^5^ responders plated with 2.0x10^5^ irradiated stimulators in growth media (RPMI media supplemented with 10% fetal bovine serum) at 37°C.

Annexin V cytotoxicity assays used cryopreserved PBMC initially collected between PID 30-60 from chimeric recipients as the responder cells, which were recovered in growth media followed by dead cell removal via bead separation. These cells were then similarly stimulated with allogenic (donor) or autologous (self) PBMC in culture. Traceable staining, stimulator irradiation, and plating were set up identical to the MLR. After 4-days, cryopreserve donor and autologous cells were recovered and depleted of dead cells as described to serve as cytotoxicity target cells. 1.0x10^5^ of these cells were stained with CFSE and introduced to their respective cultures for 4 hours. Cells were then stained with annexin V to detect apoptosis and 7-AAD for viability.

Parallel cultures were set up to evaluate intracellular granzyme B (GZMB) production and extracellular interferon-γ (IFN-γ) secretion after 4 days of stimulation. The supernatant was collected for ELISA and remaining cells were re-stimulated with PMA (81nM) and ionomycin (1.3µM) with Brefeldin A (5µg/mL) for 5 hours. Intracellular staining was performed after fixation and permeabilization for GZMB. IFN-γ ELISA was performed per manufacturers specifications with serial dilutions set up to detect low level concentrations.

### Donor-specific antibody testing

Flow cytometry-based crossmatch assays (FXM) were employed to measure pre- and post-transplant DSA levels in recipients. Plasma isolated from peripheral blood before and after transplantation was serially diluted (1:5, 1:25, 1:125, 1:625) and incubated with donor PBMC. Cells were then washed and stained. Post-transplant IgG median fluorescence intensities (MFIs) were compared to pretransplant controls on CD3+ and CD20+IgD+ cells to determine the positivity of T and B cell crossmatches, respectively.

## Results

### Chimerism induction and outcomes

**Figure 1:**
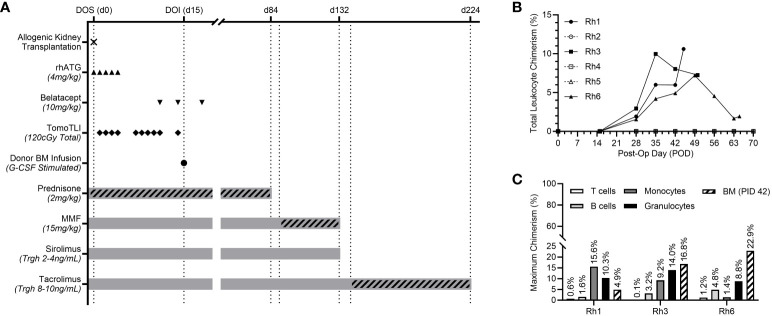
**(A)** Experimental post-transplant, TomoTLI, ATG, and belatacept-based tolerance induction protocol and immunosuppression taper schedule. **(B)** Total leukocyte chimerism kinetics for individual recipients. **(C)** Maximum chimerism levels among lineage specific PBMC subsets and CD34+ bone marrow (BM – measured PID 42).

Six rhesus macaques underwent tolerance induction in a living allogenic transplantation model. MHC disparities between the transplant pairs ranged from 2-4 MHC Class I mismatches and 0-1 MHC Class II mismatches (**Table 1**). The post-transplant, non-myeloablative tolerance induction protocol, consisting of TomoTLI, rhATG, and belatacept conditioning is shown in **Figure 1A**.

**Table 1:** Recipient MHC matching, infusion product composition, and engraftment outcomes.

	MHC Class I Ag Mismatch	MHC Class II Ag Mismatch	MHC Typing							Total Cells (x10^9/kg)	CD34+ Cells (x10^6/kg)	Chimerism (Leukocyte)
Rh1	2	0	R	A004	A074	B012b	B017a	**DR03a**	DR04a	3.0	24.7	Yes
			D	A001	A001	B047a	B047a	**DR03a**	**DR03a**			(10.6%)
Rh2	2	1	R	A025	**A004**	**B012b**	B012b	**DR04a**	DR03f	2.6	23.3	No
			D	A001	**A004**	**B012b**	B001a	**DR04a**	DR03a			
Rh3	3	1	R	A019	**A004**	B015c	B012b	**DR03a**	DR04a	3.0	18.6	Yes
			D	A001	**A004**	B047a	B048	**DR03a**	DR01a			(10.0%)
Rh4	3	1	R	A004	**A023**	B017a	B043a	**DR10a**	DR06	5.1	30.6	No
			D	A001	**A023**	B015b	B043b	**DR10a**	DR03a			
Rh5	3	1	R	A004	**A002a**	B056b	B001a	**DR15a**	DR15a	5.8	15.0	No
			D	A001	**A002a**	B055	B015a	**DR15a**	DR03g			
Rh6	4	1	R	A023	A004	B012b	B001a	**DR04a**	DR15c	1.3	14.9	Yes
			D	A001	A042	B048b	B047a	**DR04a**	DR09b			(7.2%)

Small letters: MHC subtype.

Bold: Indicating the matched MHC alleles between donor and recipient.

Recipients received 3.0-5.8x10^9^ total cells/kg and 14.9-30.6x10^6^ CD34+ cells/kg from the G-CSF-stimulated donor (**Table 1**). There was no correlation between induction of chimerism and MHC disparity nor total/CD34+ cell counts included in the infusion product.

Following donor-derived BM infusion, chimerism was established in three of six recipients (50%). Maximum leukocyte chimerism ranged from 7.2-10.6% in the peripheral immune system (**Figure 1B**). Multilineage chimerism was established in all major leukocyte subsets, though the highest levels were observed in non-lymphocyte populations, including granulocytes and monocytes (**Figure 1C**). All recipients with peripheral chimerism also demonstrated engraftment within their central BM compartment, ranging from 4.9-22.9% measured via aspirates on PID 42 (**Figure 1C**).

No chimeric animals appeared to develop alloreactivity. This was evidenced by their persistent chimerism, lack of DSA production, and allograft histology without evidence of immunologic injury. Specifically, FXM remained negative during the lifespan of all three chimeric recipients (**Supplementary Figure 1**). Further, histologic staining revealed minimal interstitial inflammation without evidence of glomerulitis, tubulitis, peritubular capillaritis or C4d positivity (**Supplementary Figure 2**). In this context, no chimeric recipients experienced allogeneic graft loss nor developed evidence of acute cellular or antibody-mediated rejection. In addition, there were no episodes of engraftment syndrome or GVHD. Of the three non-chimeric animals, two (Rh2, Rh4) experienced graft loss secondary to acute rejection early in the immunosuppression taper. Specifically, by the time of necropsy, these recipients developed 13.2-fold and 27.3-fold increases in B and T cell cross matches, respectively (**Supplementary Figure 1**). Accordingly, terminal histology for both animals demonstrated positive C4d staining indicative of DSA deposition, as well as significant mononuclear interstitial infiltration, tubulitis, and peritubular capillaritis suggesting acute cellular rejection (**Supplementary Figure 3**). The third non-chimeric animal (Rh5) was euthanized for weight loss on day 51 without evidence of rejection at the time of necropsy.

### Early immunomodulatory effects of conditioning and chimerism induction

**Figure 2:**
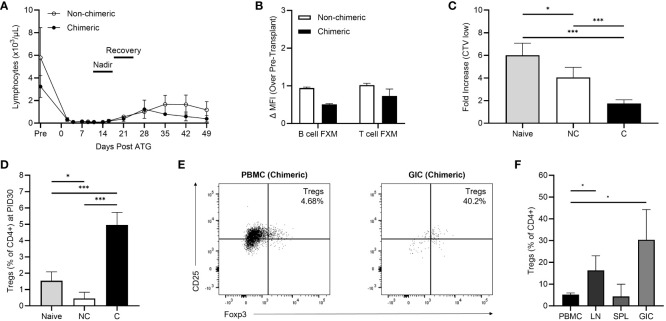
**(A)** Lymphocyte depletion and recovery kinetics between non-chimeric (n=3) and chimeric (n=3) animals. **(B)** Early post-kidney transplant (postoperative day 30-45) B cell and T flow cross match levels. **(C)** Early post-infusion (PID 30-60) allogenic MLR responses in non-chimeric (NC) and chimeric **(C)** animals compared to naïve controls. **(D)** Peripheral Treg frequencies collected on PID 30 in non-chimeric (NC) and chimeric **(C)** macaques compared to naïve controls. **(E)** Representative flow plots demonstrating levels of CD25+FoxP3+ Tregs within peripheral blood and graft infiltrating CD4+ T cells among chimeric recipients at the time of necropsy. **(F)** Percentages of Tregs present among CD4+ T cells within the peripheral blood (PBMC), draining lymph nodes (LN), spleens (SPL), and allografts (GIC) of chimeric recipients at the time of necropsy. *p < 0.05; ***p < 0.001.

The tolerance conditioning protocol yielded significant lymphocyte depletion, with an expected precipitous decline in total lymphocyte count observed by day 2 followed by a statistically significant nadir of <100/µL by day 14 (**Figure 2A**). Lymphocyte recovery (defined as a statistical increase in cell count compared to the nadir) occurred for all recipients by day 28. Similar depletion kinetics were observed for monocytes and neutrophils, though recoveries were day 21 and 35, respectively. (**Supplementary Figure 4**).

The humoral response was studied to investigate generation of post-transplant donor specific antibody. All recipients had a negative B and T-cell crossmatch prior to transplant and subsequently exhibited a negative B cell and T cell FXM in the early (day 30-45) post-transplant period regardless of chimerism induction (**Figure 2B**).

Cellular alloreactivity was studied in the early post-conditioning period via MLRs on PID 30-60 (**Figure 2C**). The allo-MLR response was defined as the fold increase in T cell proliferation against allogenic donor cells over the baseline T cell proliferation observed against autologous (self) cells. The post-transplant conditioning regimen yielded a dampened recipient allo-MLR response against donor cells compared to naïve, pre-transplant controls (n=5). Specifically, chimeric recipients (n=3) demonstrated a 70.9% decrease in the allo-MLR response compared to naïve controls (p<0.001), which correlated to a 57.0% reduction in proliferation compared to non-chimeric recipients (p<0.001). Non-chimeric recipients (n=3) exhibited a 32.6% reduction in T cell proliferation compared to naïve responses (p=0.038).

Correlative studies examining CD4+CD25+Foxp3+ Treg frequencies within the peripheral blood demonstrated elevated levels among chimeric recipients in the early post-conditioning period. By PID 30, the peripheral circulating CD4+ Treg fraction in chimeric recipients was 5.24% versus 1.54% (p<0.001) in naïve recipients, and 0.45% (p<0.001) in non-chimeric macaques (**Figure 2D**). In contrast, non-chimeric recipients harbored fewer Tregs than naïve, pre-transplant animals (p=0.024) and experienced no statistical increase in Treg frequency at later time points.

Some chimeric animals developed weight loss or Parvovirus infection requiring euthanasia according to the protocol approved by the University of Wisconsin-Madison Institutional Animal Care and Use Committee. In those animals, Tregs were able to be evaluated in the initial post-induction period within secondary lymphoid and allograft tissue (**Figures 2E, F**). Few lymphocytes were present in the non-rejecting allografts of chimeric recipients consistent with absence of a cellular allo-immune response. Though among those present, Tregs represented a considerable proportion (30.40%) of the isolated graft-infiltrating CD4+ T cells, significantly higher than the frequency observed in circulation (p=0.041). Similarly, Tregs were present at elevated frequencies (16.34%) among lymph node derived CD4+ T cells (p=0.045), though no difference was observed between PBMC and spleen. Circulating peripheral Tregs at time of necropsy remained enriched (5.17% of CD4+ T cells) compared to pre-transplant controls, with no statistical decrease in frequency compared to PID30.

### PD-1 expression during T cell homeostatic recovery

**Figure 3:**
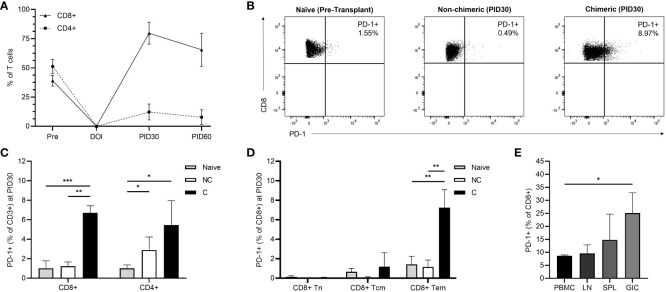
**(A)** Longitudinal post-infusion CD8+ and CD4+ T cell recovery among all recipients. **(B)** Representative flow plots demonstrating CD8+ T cell PD-1 expression among naïve, non-chimeric, and chimeric recipients at PID 30. **(C)** PD-1 expression among CD8+ and CD4+ T cells on PID 30 in non-chimeric (NC) and chimeric **(C)** animals compared to naïve controls. **(D)** PD-1 expression among CD8+ T cell subsets on PID 30 in non-chimeric (NC) and chimeric **(C)** animals compared to naïve controls. **(E)** CD8+ T cell PD-1 expression within the peripheral blood (PBMC), draining lymph nodes (LN), spleens (SPL), and allografts (GIC) of chimeric recipients at the time of necropsy. *p < 0.05; **p < 0.01; ***p < 0.001.

Longitudinal T cell immunophenotyping was performed during homeostatic recovery for all recipients. The TomoTLI induction protocol was associated with an inversion of peripheral blood CD8:CD4 polarization compared to pre-transplant controls irrespective of engraftment (**Figure 3A**). By PID 30, circulating CD8+ T cells were predominant, comprising 64.7% of the T cell compartment, nearly double the frequency observed among naïve controls (35.4%, p=0.013). Conversely, a minority of circulating T cells were CD4+ by PID 30, which were decreased 4-fold compared to pre-transplant controls (11.9% vs 46.1%, p<0.001).

Among circulating CD8+ T cells, the CD28-CD95+ effector memory (Tem) phenotype was predominant at 94.13%, followed by the CD28+CD95+ central memory (Tcm) subset at 4.29%, and the CD28+CD95- naïve (Tn) subset at 0.69% (**Supplementary Figure 5**). There were no differences in CD8+ Tem, Tcm, and Tn frequencies between chimeric and non-chimeric recipients.

Though longitudinal T cell subset frequencies were similar regardless of whether chimerism was achieved, PD-1 expression on emerging circulating CD3+CD8+ T cells was significantly higher among chimeric recipients than both non-chimeric hosts and naïve controls (p<0.001). In contrast, no upregulation of PD-1 expression was observed in CD8+ T cells among non-chimeric recipients following conditioning, nor was there a statistical increase over time. Specifically, by PID 30, CD8+PD-1+ T cells represented 6.49% of circulating T cells in chimeric hosts, which was a 6.42-fold increase compared to naïve animals (1.01%, p<0.001) and a 5.27-fold increase compared to that observed in non-chimeric recipients (1.23%, p=0.001) (**Figures 3B, C**). At the same timepoint (PID 30), CD4+ PD-1 expression was elevated compared to naïve controls in both chimeric (5.44%, p=0.028) and non-chimeric (2.89%, p=0.040) recipients, though there was no statistical difference between the two recipient groups (**Figure 3C**).

Further evaluation within the CD3+CD8+ compartment demonstrated significant upregulation of PD-1 expression among the Tem phenotype in chimeric recipients (7.25%) compared to naïve (1.41%, p=0.002) and non-chimeric (1.16%, p=0.006) macaques (**Figure 3D**). Conversely, CD8+ central memory and naïve phenotypes demonstrated similar frequencies of PD-1 expressing cells irrespective of engraftment. There was no difference in PD-1 expression among any CD4+ subset between the chimeric and non-chimeric groups.

At the time of necropsy, PD-1 expression was evaluated among T cells isolated from circulation, secondary lymphoid tissue, and grafts of chimeric recipients (**Figure 3E**). CD8+ T cell PD-1 upregulation persisted within the peripheral circulation and was similar to that observed on PID30. Though low numbers of cells were isolated, PD-1 expression was elevated among CD8+ T cells within the graft compared to circulating PBMC (25.10% vs 8.68%; p=0.028). There were similar relative levels of CD8+ PD-1 expression within the peripheral circulation, lymph nodes (9.58%) and spleen (14.79%). No difference in PD-1 expression was detected among CD4+ T cells isolated from PBMC, lymph nodes, spleen, or the allograft.

### Functional implications of PD-1 expression in the early alloreactive T cell response

**Figure 4:**
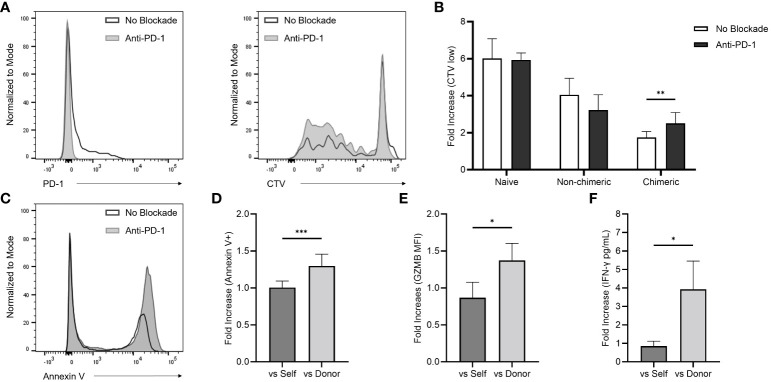
**(A)** Flow histograms demonstrating the effects of *in vitro* PD-1 blockade on PBMC-derived CD8+ T cell PD-1 expression (left) and total T cell proliferation represented by CTV low populations (right). **(B)** Early post-infusion (PID 30-60) allogenic MLR responses with and without the addition of the anti-PD-1 agent among naïve, non-chimeric, and chimeric macaques. **(C)** Representative flow histogram demonstrating the effect of *in vitro* PD-1 blockade on target cell annexin V positivity in cytotoxicity assays performed with chimeric recipient PBMC (collected between PID 30-60) targeted against allogenic donor PBMC. **(D)** Fold-increase in target cell annexin V positivity in cytotoxicity assays performed with self- and donor-simulated chimeric recipient PBMC (collected between PID 30-60) exposed to *in vitro* PD-1 inhibition over non-inhibited controls. **(E, F)** Fold-increase self- and donor-stimulated intracellular GZMB expression and extracellular IFN-γ with the addition of the *in vitro* anti-PD-1 agent using Rh6 PBMC collected at multiple post-transplant timepoints over non-inhibited controls.

The role of PD-1 expression in the early immune environment generated by the mixed-chimeric state was examined by introducing a rhesus anti-PD-1 blocking agent to *in vitro* alloreactivity assays. Functional assays were performed on PBMC collected during the early post-conditioning period (PID 30-60). In the allogenic MLR, inhibition of PD-1 uncovered a 1.43-fold increase (p=0.004) in T cell proliferation compared to the physiologic control (without PD-1 blockade) among all three chimeric recipients (**Figures 4A, B**). In contrast, T cells from naïve (n=5) and non-chimeric (n=3) macaques did not demonstrate higher proliferative responses with the addition of anti-PD-1.

Cytotoxicity assays were performed using cells collected from chimeric recipients (n=3) during homeostatic recovery (**Figures 4C, D**). These cells were stimulated over four days in culture using irradiated recipient (autologous) or donor (allogenic) cells before introducing target cells to their respective cultures. Consistent with the MLR data, *in vitro* PD-1 inhibition uncovered a 1.3-fold increase in cytotoxic activity against donor cells compared to non-blockade controls, an effect that was not observed against autologous targets (p<0.001).

In a parallel experiment, intracellular GZMB and extracellular IFN-γ production were analyzed after four days of *in vitro* stimulation (**Figures 4E, F**). PBMC samples from one chimeric recipient (Rh6) collected at multiple timepoints were tested. PD-1 blockade yielded a 1.6-fold increase in GZMB MFI versus physiologic, non-blockade controls, which was not observed with the addition the anti-PD1 blockade to cultures stimulated with autologous cells (p=0.018). In a similar way, ELISA detection revealed higher levels (4.6-fold) of IFN-γ secretion in the donor-stimulated supernatants exposed to the anti-PD-1 blocking agent, though no change in IFN-γ secretion was elicited in the autologous cultures (p=0.026).

## Discussion

We have demonstrated that mixed chimerism can be achieved in a MHC-disparate rhesus macaque BM-derived HC and kidney transplant model using nonmyeloablative conditioning consisting of TomoTLI, ATG, and belatacept therapies. Importantly, in previous reports we have shown that transient mixed chimerism generated by this tolerance induction method is capable of inducing sustained, long-term allograft survival off all immunosuppression (17). Though no chimeric recipients in this study cohort survived to IS withdrawal, there was no evidence of clinical alloreactivity at the time of necropsy, as indicated by lack of DSA production and absence of immunologic injury on histology.

In this study we sought to better characterize the early (60 days post-transplant) immunologic effects of mixed chimerism induction. We observed that early anti-MHC humoral immune responses were controlled with the addition of T cell co-stimulation blockade (belatacept) to the conditioning regimen. With this modified protocol, all recipients demonstrated negative B cell and T cell FXMs in the early post-transplant period, which was associated with an increased rate of engraftment to 50% among the widely MHC disparate pairs in this study. These results contrasted with our previous findings in the 1-haplotype matched group that received the same Tomotherapy tolerance induction protocol but without belatacept. In that series of animals, development of *de novo* DSA occurred in all recipients except the 11% that achieved transient mixed chimerism and long-term tolerance (17). These observations are consistent with the premise that belatacept provided improved control of the early post-transplant humoral response at the time of bone marrow transplantation and improved central engraftment of donor cells.

In addition, and consistent with the prior protocol, we observed that conditioning efficiently depleted peripheral leukocytes, with all lineages reaching a nadir prior to infusion of the allogenic HC product. Further, MLRs performed in the early post-infusion period revealed a markedly diminished allogenic T cell response in chimeric animals compared to naïve controls. Taken together, these findings demonstrated that TomoTLI, ATG, and belatacept-based conditioning was effective at controlling early humoral and cellular alloreactivity, thus creating a favorable immunologic environment to support HC engraftment and mixed chimerism.

Though establishment of mixed chimerism is a proven strategy for induction of allograft tolerance after kidney transplantation, the necessary magnitude and duration of chimerism remains unknown. We have demonstrated that low level, transient mixed chimerism was sufficient for the generation of important peripheral mechanisms involved in cellular immunomodulation. One mechanism involved expansion of regulatory T cells, which are known to be important mediators of allogenic tolerance after induction of transient mixed chimerism. Several studies have shown that Tregs are highly enriched during homeostatic recovery and are directly involved in modulation of T cell alloreactivity (18, 19, 31–34). Accordingly, we found that circulating Tregs were expanded during post-infusion immune recovery within chimeric hosts compared to naïve and non-chimeric macaques. Further, assessment of allograft-resident cells at the time of necropsy revealed marked Treg enrichment, which was also observed in draining secondary lymphoid tissue. This is consistent with prior reports showing that graft infiltrating and tumor resident Tregs are antigen-specific and present at higher frequencies within tolerant tissue than in immunoreactive grafts (35–37). These findings indicated that donor cell engraftment promoted a phenotypic shift toward T cell regulation in the early, post-transplant peripheral immune system and suggested that Treg homing to sites of alloantigen-rich environments may contribute to the establishment of peripheral tolerance.

PD-1 is a co-inhibitory molecule expressed on emerging T cells during thymic education, as well as mature CD4+ and CD8+ T cells in the periphery (38–43). When engaged with its ligand, PD-L1, it drives T cell exhaustion and antigen-specific hypo-reactivity during the development of central and peripheral tolerance (44, 45). In the periphery, PD-1 signaling reduces effector differentiation and expansion while also inducing antigen-specific T cell exhaustion among terminally differentiated phenotypes, thus controlling auto- and alloreactivity (46–54). Among chimeric recipients of allogenic bone marrow, PD-L1 upregulation on non-hematopoietic populations serves to restrict effector T cell-mediated damage, thus promoting tolerance (40, 47, 55, 56). In a similar way, PD-L1 expression on allogenic cells during islet transplantation has been found to be protective against rejection (47, 57). Furthermore, when treated with PD-1 inhibitors for oncologic therapy, renal transplant recipients experience significantly higher rates of allograft rejection (58, 59). Of particular relevance to this study, Haspot et al. demonstrated that tolerance induction after allogenic bone marrow transplantation was dependent on PD-1 upregulation and signaling among CD8+ T cells within the periphery (60). Collectively, these findings underscore the importance of PD-1 co-inhibition in transplant immunology and control of CD8+ T cell alloreactivity.

In our model we found a predominance of circulating CD8+ T cells within the emerging immune system, in contrast to the relatively low frequency of CD4+ T cells. This CD8 polarity was observed in both non-chimeric and chimeric recipients, suggesting that conditioning, rather than engraftment, was driving this pattern of T cell recovery. However, there was an upregulation of PD-1 on the surface of emerging CD8+ T cells in chimeric animals only, which was most prominently expressed within the effector memory subset. Similar to Tregs, these PD-1 upregulated cells were found at higher concentrations within the graft, further supporting a local immunoregulatory environment after induction of mixed chimerism. Importantly, its role in early alloreactivity was supported by an augmented donor-specific T cell response with the addition of an anti-PD-1 blocking agent to post-transplant allogenic MLRs performed with chimeric recipient cells. This effect was not observed when naïve and non-chimeric allo-MLRs were exposed to an *in vitro* PD-1 blockade, indicating that the increased T cell proliferative response is specific to an underlying allo-protective role of PD-1 in chimeric animals.

To better evaluate the implications of PD-1 expression in CD8+ T cell-mediated alloreactivity, cytotoxicity assays were performed in which PD-1 blockade similarly exposed an allogenic response but had no effect against autologous controls. Further, PD-1 inhibition in chimeric recipient cells yielded increased excretion and production of the CD8+ T cell effector molecules, IFN-γ and GZMB. Taken together, these findings suggest that post-engraftment CD8+ T cell co-inhibition via PD-1 signaling plays an important allo-protective role and is involved in the establishment of mixed chimerism-based operational tolerance.

This mechanistic study of the early immunomodulation associated with mixed chimerism induction demonstrated that non-myeloablative, TLI-based conditioning with ATG and belatacept dampened humoral and cellular allogenic responses and is capable of supporting HC engraftment in widely MHC disparate transplantation. Specifically, these findings demonstrated that low level donor immune cell engraftment was associated with homeostatic expansion of Tregs and upregulation of PD-1 expression among emerging recipient CD8+ T effector memory cells, both of which contributed to allogenic immunomodulation. In conjunction with our prior studies, we posit that these early immune cell mechanisms may contribute to the establishment of TLI-based, mixed chimerism-induced operational tolerance and serve as important foundational knowledge for future protocols.

## Data availability statement

The raw data supporting the conclusions of this article will be made available by the authors, without undue reservation.

## Ethics statement

The animal study was approved by University of Wisconsin-Madison Institutional Animal Care and Use Committee. The study was conducted in accordance with the local legislation and institutional requirements.

## Author contributions

CL: Conceptualization, Data curation, Formal analysis, Investigation, Methodology, Project administration, Resources, Supervision, Validation, Visualization, Writing – original draft, Writing – review & editing. SK: Conceptualization, Data curation, Formal analysis, Funding acquisition, Investigation, Methodology, Project administration, Resources, Supervision, Validation, Writing – review & editing. JF: Data curation, Methodology, Project administration, Resources, Supervision, Writing – review & editing. JP: Methodology, Project administration, Resources, Supervision, Writing – review & editing. JC: Project administration, Resources, Supervision, Writing – review & editing. PC: Data curation, Methodology, Resources, Writing – review & editing. MW: Data curation, Investigation, Writing – review & editing. DK: Data curation, Investigation, Writing – review & editing. SS: Conceptualization, Project administration, Resources, Writing – review & editing. DK: Conceptualization, Funding acquisition, Methodology, Project administration, Resources, Supervision, Validation, Writing – review & editing.
