# Supplementary material for: Early allogeneic immune modulation after establishment of donor hematopoietic cell-induced mixed chimerism in a nonhuman primate kidney transplant model
Source: Front Immunol. 2024 Jan 22;15:1343616. doi: 10.3389/fimmu.2024.1343616 (PMC10839019; doi:10.3389/fimmu.2024.1343616)
Supplement: Supplementary file 1 [file DataSheet_1.docx]

**SUPPLEMENTAL MATERIALS**

| **Antibody Target / Reagent** | **Fluorochrome** | **Clone** | **Manufacturer** |
| --- | --- | --- | --- |
| **Chimerism Panel** | | | |
| CD45 | FITC | D058-1283 | BD Biosciences, San Jose, CA |
| CD3 | PerCP-Cy5.5 | SP34-2 | BD Biosciences, San Jose, CA |
| Mamu-A*01 | PE | P12 | NIH Nonuman Primate Reagent Resource, Boston, MA |
| CD8 | PE-Cy7 | RPA-T8 | BD Biosciences, San Jose, CA |
| CD20 | BUV395 | 2H7 | BD Biosciences, San Jose, CA |
| CD14 | BV421 | M5E2 | BioLegend, San Diego, CA |
| CD4 | BV650 | L200 | BD Biosciences, San Jose, CA |
| HLA-DR | APC | L243 | BioLegend, San Diego, CA |
| CD11b | APC-Cy7 | ICRF44 | BD Biosciences, San Jose, CA |
| **Tcell Immunophenotyping** | | | |
| PD-1 | Alexa488 | EH12.2H7 | BioLegend, San Diego, CA |
| CD95 | PerCP-Cy5.5 | DX2 | BioLegend, San Diego, CA |
| CD25 | PE-Cy7 | BC96 | BioLegend, San Diego, CA |
| CD3 | BUV395 | SP34-2 | BD Biosciences, San Jose, CA |
| CD8 | BUV496 | RPA-T8 | BD Biosciences, San Jose, CA |
| Live/Dead Aqua | Aqua |  | Thermo Fisher Scientific, Waltham, MA |
| CD4 | BV650 | L200 | BD Biosciences, San Jose, CA |
| FOXP3 | APC | PCH101 | Thermo Fisher Scientific, Waltham, MA |
| CD28 | APC-Cy7 | CD28.2 | BioLegend, San Diego, CA |
| **MLR** | | | |
| Live/Dead Green | Green |  | Thermo Fisher Scientific, Waltham, MA |
| GZMB | PE | GB11 | BD Biosciences, San Jose, CA |
| CD3 | BUV395 | SP34-2 | BD Biosciences, San Jose, CA |
| CD8 | BUV496 | RPA-T8 | BD Biosciences, San Jose, CA |
| CTV | CellTrace Violet |  | Thermo Fisher Scientific, Waltham, MA |
| CD4 | BV605 | L200 | BD Biosciences, San Jose, CA |
| CTFR | CellTrace Far Red |  | Thermo Fisher Scientific, Waltham, MA |
| **Cytotoxicity** | | | |
| CFSE | CFSE |  | Thermo Fisher Scientific, Waltham, MA |
| Annexin V | PE |  | Thermo Fisher Scientific, Waltham, MA |
| 7-AAD | 7-AAD |  | Thermo Fisher Scientific, Waltham, MA |
| CTV | CellTrace Violet |  | Thermo Fisher Scientific, Waltham, MA |
| CTFR | CellTrace Far Red |  | Thermo Fisher Scientific, Waltham, MA |
| **Flow Cross Match** | | | |
| IgG | FITC | 1B3 | NIH Nonuman Primate Reagent Resource, Boston, MA |
| CD3 | PE | SP34-2 | BD Biosciences, San Jose, CA |
| CD20 | PE-Cy7 | 2H7 | BioLegend, San Diego, CA |
| IgD | Alexa647 | AF647 | SouthernBiotech, Birmingham, AL |

**Supplemental Table 1.** Flow cytometric panels, antibody clones, and reagent manufacture

**Supplemental Figure 1.** B cell and T cell flow cross match (FXM) data from early post-transplant (POD 30-45) vs terminal (at time of necropsy) timepoints among chimeric and non-chimeric recipients.


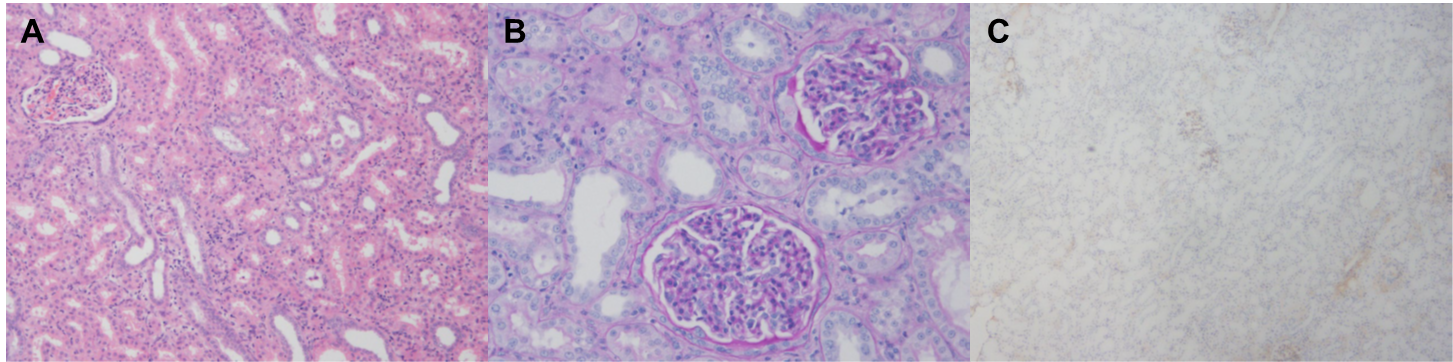


**Supplemental Figure 2.** Representative hematoxylin and eosin (A), periodic acid-Schiff (B) and C4d (C) staining for chimeric recipients demonstrating minimal interstitial inflammation without evidence of glomerulitis, tubulitis, peritubular capillaritis, or C4d positivity suggesting no development of acute cellular or antibody-mediated rejection.


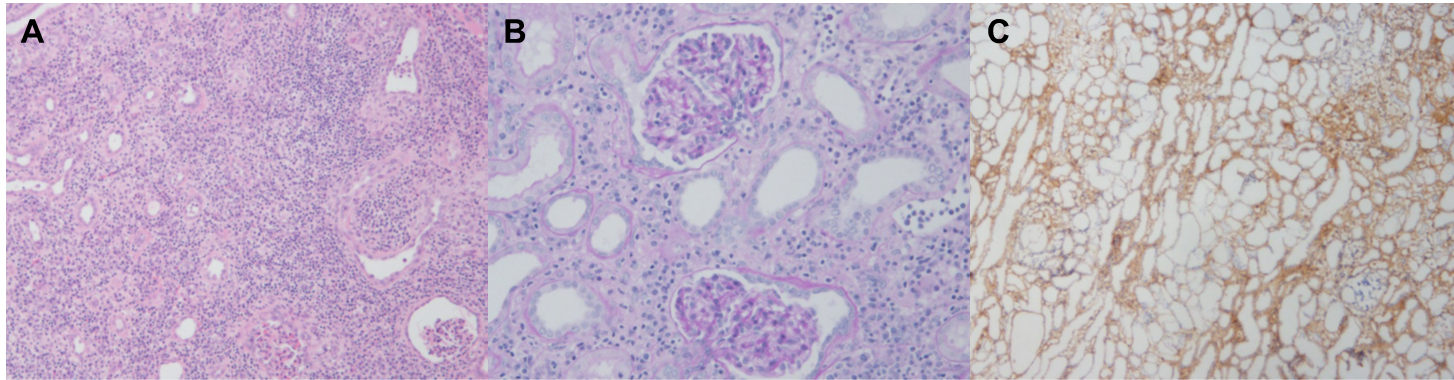


**Supplemental Figure 3.** Representative hematoxylin and eosin (A), periodic acid-Schiff (B) and C4d (C) staining for non-chimeric recipients demonstrating significant mononuclear interstitial infiltration, tubulitis, peritubular capillaritis, and C4d positivity suggesting acute cellular and antibody-mediated rejection.


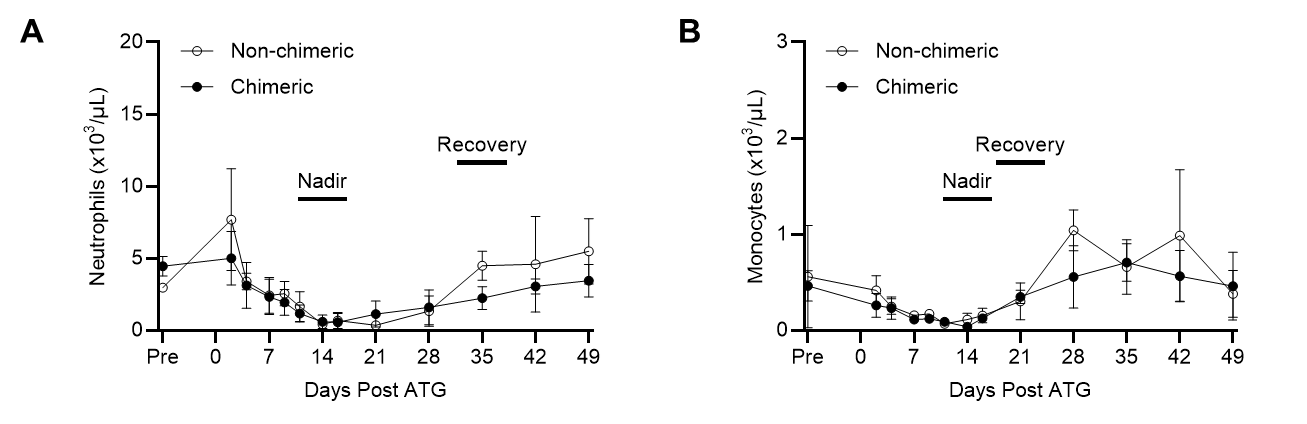


**Supplemental Figure 4.** Neutrophil (A) and monocyte (B) depletion and recovery kinetics


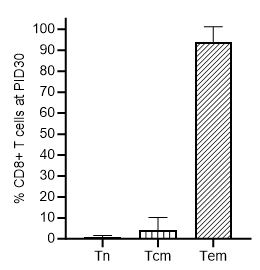


**Supplemental Figure 5.** CD8+ T cell subset immunophenotyping at PID 30.
